# Clinical Relevance of Gastroesophageal Cancer Associated SNPs for Oncologic Outcome After Curative Surgery

**DOI:** 10.1245/s10434-021-10771-y

**Published:** 2021-09-16

**Authors:** Jin-On Jung, Naita Maren Wirsik, Henrik Nienhüser, Leila Peters, Beat Peter Müller-Stich, Timo Hess, Vitalia Schüller, Johannes Schumacher, Thomas Schmidt

**Affiliations:** 1 Department of General, Visceral and Transplantation Surgery, University of Heidelberg, Heidelberg, Germany; 2 Institute of Human Genetics, University of Bonn, Bonn, Germany; 3 Institute of Human Genetics, University of Marburg, Marburg, Germany; 4 Present Address: Present address: Department of General, Visceral and Transplantation Surgery, University Clinic Cologne, Cologne, Germany

## Abstract

**Background:**

Gastric and esophageal cancers are malignant diseases with rising importance in Western countries. To improve oncologic outcome after surgery, it is essential to understand the relevance of germline mutations. The aim of the study was to identify and distinguish clinically relevant single-nucleotide polymorphisms (SNPs).

**Patients and Methods:**

In total, 190 patients with curative oncological resections of gastric and distal esophageal adenocarcinomas at Heidelberg University Hospital were eligible for this study. Outcome differences were determined for each SNP by analysis of clinical variables, survival, and mRNA expression levels.

**Results:**

Significant survival differences were found on univariate analysis for usual prognostic variables (such as pTNM) and for six SNPs. On multivariate survival analysis, the SNPs rs12268840 (intron variant of *MGMT*, *p* = 0.045) and rs9972882 (intron variant of *STARD3* and eQTL of *PGAP3*, *p* = 0.030) were independent and significant survival predictors along with R status and pT/pN category. Group TT of rs12268840 had the highest rate of second primary carcinoma (30.4%, *p* = 0.0003), lowest expression of *MGMT* based on cis-eQTL analysis in normal gastroesophageal tissue (*p* = 1.99 × 10^−17^), and worst oncologic outcome. Group AA of rs9972882 had the highest rate of distant metastases pM1 (42.9%, *p* = 0.0117), highest expression of *PGAP3* (*p* = 1.29 × 10^−15^), and worst oncologic outcome.

**Conclusions:**

Two intron variant SNPs of *MGMT* and *STARD3* were identified that were significant survival predictors and may influence tumor biology. The data indicate that DNA methylation (*MGMT*) and malfunction of GPI anchoring (*PGAP3*) are distinct mechanisms that are relevant for tumor progression and relapse.

**Supplementary Information:**

The online version contains supplementary material available at 10.1245/s10434-021-10771-y.

Cancers of the stomach and esophagus are devastating diseases accounting for nearly 9% of all malignancies.^1^ In Western countries, more than two-thirds of gastric cancer cases are diagnosed at an advanced stage with a poor 5-year survival rate of about 25%.^2^ Moreover, the incidence of esophageal adenocarcinoma has been rapidly increasing.^3^ Gastroesophageal reflux disease (GERD) and Barrett’s esophagus (BE) apart from obesity and tobacco smoking were identified as significant risk factors,^4^ especially for adenocarcinoma of the gastroesophageal junction (GEJ). Preexisting GERD leads to a 6.2-fold risk for GEJ,^3^ while antireflux therapies, physical activity, breastfeeding, nonsteroidal antiinflammatory drugs (NSAIDs), and HMG-CoA reductase inhibitors (statins) are inversely associated risk factors. Similar risk factors could be identified for gastric cancer (GC) as GERD bears a two- to fourfold risk for GC.^5^ Furthermore, it is known that chronic infection with *Heliobacter pylori* is another risk for GC.

Genome-wide genotyping is a frequently used technique that has been widely practiced in the past. Recently, various single-nucleotide polymorphisms (SNPs) were identified that associate with either GERD, BE, GEJ, or GC.^6^ Most studies utilize high case numbers and hundreds of thousands of SNPs, but do not address specific clinical parameters other than the rough differentiation between case and control groups. However, it is necessary to establish ties between genotyped data and oncological outcome by making use of systematic follow-up on a well-differentiated cohort. While the number of allegedly relevant SNPs increases, it becomes harder to maintain an overview of those SNPs, which might also have a clinical significance and lead back to molecular pathways that are actually relevant. By correlating genotyped and imputed data with oncologic outcome parameters, we aim to identify clinically relevant loci.

## Patients and Methods

### Data Collection

Eligible subjects of this study were patients with GC or GEJ who underwent oncological resection of gastric or esophageal cancer between 2008 and 2017 at Heidelberg University Hospital, Department of General Surgery. All patients provided written consent for data collection and analysis. Patients were excluded who deceased during the early postoperative phase or in-hospital as oncologic outcome was assessed. The trial protocol was approved by the ethics committee at the University of Heidelberg and was performed in accordance with the Declaration of Helsinki and Good Clinical Practices as well as local ethics and legal requirements.

### Genotyping

Prior to oncological resection, genomic DNA was extracted from peripheral blood leukocytes. Genotyping was performed by collaborating partners at the Department of Human Genetics, University of Bonn, Germany. It was accomplished for all patients using Infinium OmniExpress, Infinium OmniExpressExome, and Infinium Omni2.5Exome BeadChips (by Illumina) according to the manufacturer’s protocol at the University of Bonn. The overlap of the SNP content was subjected to initial quality control performed with PLINK v1.90b6.6. Samples with genotype call rate less than 97%, discrepancies in sex, divergent ancestry from the CEU HapMap 2010 population, and related samples were excluded from further analysis.

### Imputation

All unambiguous SNPs with SNP call rate >95%, minor allele frequency (MAF) >1%, and Hardy–Weinberg equilibrium (HWE) > 0.001 were used for imputation. Imputation was performed with IMPUTE2 based on 1,000 Genomes Phase 3 as reference panel. As post-imputation quality control, we excluded all variants with an information score (INFO-score) < 0.8, HWE < 0.001, MAF < 1%, and SNP missing rate > 5% for best-guessed genotypes at posterior probability greater than 0.9 from further analysis.

### Histopathology and Follow-Up

In all cases, the diagnosis of gastric and esophageal adenocarcinoma was histopathologically confirmed and the resected specimens were analyzed at the Department of Pathology, University of Heidelberg. The histopathological staging was performed according to the 8th UICC edition, including TNM categories, R status, and grading.^7^ In-hospital mortality and postoperative complications were recorded during hospital course. To acquire long-term survival data, all patients were systematically followed up via continuous surveys. The relevant survival data for this patient collective were gathered until October 2020.

### Statistical Analysis

We extracted the genotype information from the initial PLINK files by using Gtool v0.7.5. The data were collocated using Microsoft Office Excel, and statistical tests as well as plots were calculated thereafter via GraphPad Prism 9 for Mac OS X. We defined overall survival from time of first diagnosis until death or until the most recent follow-up using the Kaplan–Meier method. Differences in survival were analyzed via Mantel–Cox (logrank) comparisons on STATA/SE 15.0 for Mac. A level of significance of *α* = 0.05 was generally considered as statistically significant. For multivariate survival analysis, we established a Cox proportional hazards model with a level of significance of *α* < 0.2. All clinical and SNP parameters from the univariate studies with a *p*-value < 0.2 were included. The preoperatively assessed cTNM categories were excluded since a precise histopathology was available via the final pTNM histology.

### Literature-Based SNP Identification

We performed a MEDLINE search via PubMed for SNPs that were reported as being responsible for or associated with BE, GERD, GEJ, or GC in previous genome-wide association studies. Search items varied and included terms such as “genome wide association study,” “gastric cancer,” “esophageal cancer,” “gastroesophageal reflux,” “Barrett’s esophagus,” “SNP,” and others. As of January 2021, a MeSH search with terms “genome wide association study” in combination with either “gastric neoplasm” (*n* = 122, 42%), “esophageal neoplasm” (*n* = 117, 40%), “gastroesophageal reflux” (*n* = 13, 4%), or “Barrett’s esophagus” (*n* = 41, 14%) showed a total result of 293 original articles. All reported SNPs were searched, and those that were available in the genotyped and imputed datasets were further analyzed.

### cis-eQTL Analysis in Normal Tissue

For cis expression quantitative trait loci (cis-eQTL) analyses in normal tissue, we accessed the Genotype-Tissue Expression (GTEx) database (Version 8; Release date 18 July 2019: dbGaP Accession phs000424.v8.p2; http://www.gtexportal.org/home/). All local genes +/− 1 Mb of the selected SNPs were extracted based on data provided by the GENCODE project. We retrieved the summary statistics of linear regression results via GTEx eQTL Calculator and generated violin plots via GTEx eQTL Dashboard with layout modifications via Adobe Illustrator CC 2018 (Version 22.0.0).

## Results

### Comparative Study—Whole Collective, GC, and GEJ

In total, 61 out of 190 patients (32.1%) were diagnosed with GEJ (Siewert type I–III) and 129 patients (67.9%) had GC. Among GEJ patients, 82.0% were male, as opposed to 58.1% of GC patients (*p* = 0.0017). GEJ patients had a higher mean BMI of 26.9 kg/m^2^ compared with GC patients (mean BMI 25.3 kg/m^2^, *p* = 0.0196). Besides neoadjuvant therapy (85.2% in GEJ versus 57.4% in GC, *p* = 0.0001), there were no significant differences between GEJ and GC patients regarding usual survival predictors. The results are demonstrated in Table 1 for the whole collective and separately for GEJ and GC in comparison, showing the significant variables that were also used in the latter multivariate survival analysis. It is important to note that both tumor entities GEJ and GC did not show any significant differences in overall survival (*p* = 0.9417) and disease-free survival (*p* = 0.3893). The corresponding Kaplan–Meier curves are demonstrated in Supplementary Fig. 1a, b.

**Table 1:** Selection of clinical survival predictors and comparison between GC and GEJ

		Whole collective (GEJ and GC)	Gastroesophageal junction cancer (GEJ)	Gastric cancer (GC)	*p*-value
Tumor entity	GEJ I/II GEJ III GC	48 (25.2%) 13 (6.8%) 129 (67.9%)	*n* = 61 (32.1%)	*n* = 129 (67.9%)	–
Sex**	Male Female	125 (65.8%) 65 (34.2%)	50 (82.0%) 11 (18.0%)	75 (58.1%) 54 (41.9%)	0.0017
BMI (in kg/m^2^)*	Mean 95% CI	25.8 25.2–26.5	26.9 25.6–28.2	25.3 24.6–26.0	0.0196
Neoadjuvant (R)CTx***	No Yes	64 (33.7%) 126 (66.3%)	9 (14.8%) 52 (85.2%)	55 (42.6%) 74 (57.4%)	0.0001
(y)pT category	0 1a 1b 2 3 4a 4b	10 (5.3%) 12 (6.3%) 19 (10.0%) 22 (11.6%) 91 (47.9%) 22 (11.6%) 14 (7.4%)	5 (8.2%) 1 (1.6%) 5 (8.2%) 7 (11.5%) 36 (59.0%) 6 (9.8%) 1 (1.6%)	5 (3.9%) 11 (8.5%) 14 (10.9%) 15 (11.6%) 55 (42.6%) 16 (12.4%) 13 (10.1%)	0.6465
(y)pN category	0 1 2 3a/b	78 (41.1%) 28 (14.7%) 37 (19.5%) 47 (24.7%)	24 (39.3%) 9 (14.8%) 16 (26.2%) 12 (19.7%)	54 (41.9%) 19 (14.7%) 21 (16.3%) 35 (27.1%)	0.9096
(y)pM category	0 1	161 (84.7%) 29 (15.3%)	52 (85.2%) 9 (14.8%)	109 (84.5%) 20 (15.5%)	0.8953
R status	0 Any X/1/2	152 (80.0%) 38 (20.0%)	46 (75.4%) 15 (24.6%)	106 (82.2%) 23 (17.8%)	0.3318
Overall survival†	Median (months) 95% CI	54.4 39.9–90.2	57.0 27.8–incalc.	52.1 38.9–94.8	0.9417
Disease-free survival†	Median (months) 95% CI	106.1 47.1–incalc.	incalc. 15.7–incalc.	106.1 51.4–incalc.	0.3893

^†^According to logrank test

^*^p < 0.05

^**^p < 0.01

^***^p < 0.001

### Overall Survival—Clinical Parameters

Median follow-up duration was 49.08 months (73.25 months for living cases and 20.74 months for deceased cases). The preoperative variables cT (*p* = 0.0045), cN (*p* = 0.0079), and cM category (*p* < 0.0001) were significant predictors of survival. Neoadjuvant therapy (*p* = 0.0229) and type of operation (*p* < 0.0001) were also clinical variables that were shown to have statistical significance. Finally, the histopathological parameters pT (*p* < 0.0001), pN (*p* < 0.0001), pM (*p* < 0.0001), and R status (*p* < 0.0001) were significant for outcome. All results are summarized in Supplementary Table 1A,B.

### Overall and Disease-Free Survival—SNPs

In total, 68 SNPs, which were identified in the literature search, were analyzed by univariate survival analysis (Supplementary Table 2A,B). Six SNPs showed significant differences on univariate overall survival (OS) analysis. The SNP IDs, genetic information, and median survival time (in months) are summarized in Table 2.

**Table 2:** SNPs with significant differences for overall survival on univariate testing, sorted by chromosome position

SNP ID	Associated gene	Position	*p*-value†	*χ*^2^†	Group 1, median survival [95% CI]	Group 2, median survival [95% CI]	Group 3, median survival [95% CI]	Refs.
rs12724079	*ASH1L*	Chromosome 1 155,464,151	0.0244	7.43	CC (*n* = 86),50.5 months [29.7–94.8 months]	TC (*n* = 74), incalc. [51.6 months–incalc.]	TT (*n* = 30), 30.2 months [19.0–56.8 months]	^25^
rs1870377	*VEGFR2*	Chromosome 4 55,106,807	0.0416	6.36	AA (*n* = 14), incalc. [50.2 months–incalc.]	TA (*n* = 57), 62.1 months [26.4 months–incalc.]	TT (*n* = 110), 50.2 months [32.1–89.9 months]	^26^
rs2898290	*LINC00208*	Chromosome 8 11,576,400	0.0428	6.30	CC (*n* = 56), 50.2 months [24.0 months–incalc.]	TC (*n* = 99), 94.8 months [50.2 months–incalc.]	TT (*n* = 35), 30.5 months [20.3–58.9 months]	^27^
rs12268840*	*MGMT*	Chromosome 10 129,527,035	0.0119	8.86	CC (*n* = 101), 62.1 months [36.0–94.8 months]	TC (*n* = 65), incalc. [39.0 months–incalc.]	TT (*n* = 23), 29.7 months [18.3–42.3 months]	^15^
rs9972882*	*STARD3*	Chromosome 17 39,651,445	0.0214	7.69	AA (*n* = 14), 20.3 months [8.2–52.1 months]	AC (*n* = 65), 85.6 months [39.5 months–incalc.]	CC (*n* = 111), 57.0 months [38.0 months–incalc.]	^28^
rs10423674	*CRTC1*	Chromosome 19 18,707,093	0.0258	7.31	AA (*n* = 18), incalc. [52.1 months–incalc.]	AC (*n* = 83), 62.1 months [38.9 months–incalc.]	CC (*n* = 89), 39.0 months [27.0–89.9 months]	^29^

^†^According to logrank test

^*^p < 0.05

**Fig. 1:**
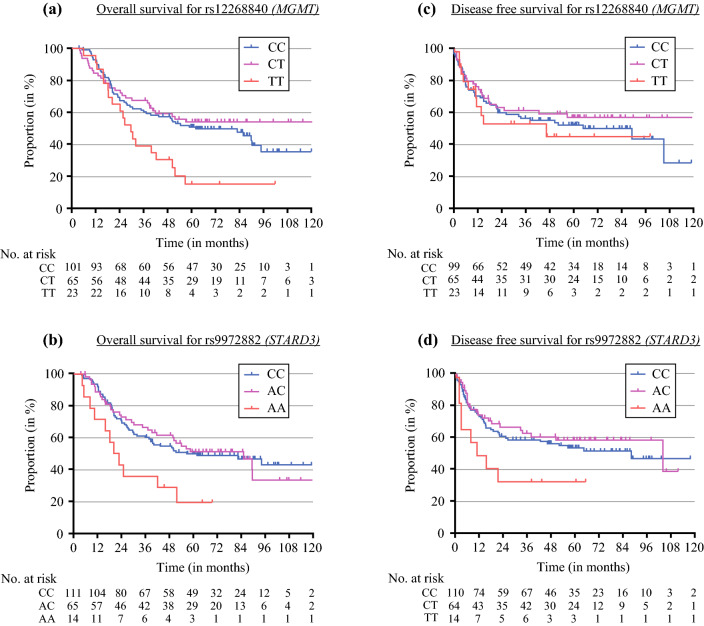
**a**, **b** Kaplan–Meier overall survival plots for groups CC/CT/TT in rs12268840 (intron variant of *MGMT*) and for groups CC/AC/AA in rs9972882 (intron variant of *STARD3*). **c**, **d** Kaplan–Meier disease-free survival plots for groups CC/CT/TT in rs12268840 and for groups CC/AC/AA in rs9972882

For rs12268840 (located on chromosome 10, intron variant of *MGMT*), 101 patients were genotyped as CC with a median OS of 62.1 months, 65 patients as TC (median OS incalculable), and 23 patients as TT with a median OS of 29.7 months (*p* = 0.0119; Fig. 1a). SNP rs9972882 is located on chromosome 17 and an intron variant of the Star-related lipid transfer domain containing 3 (*STARD3*). For rs9972882, 14 patients were genotyped as AA with a median OS of 20.3 months, 65 patients as AC with a median OS of 85.6 months, and 111 patients as CC with a median OS of 57 months (*p* = 0.0214; Fig. 1b). The Kaplan–Meier plots of the remaining four SNPs with significant survival differences in univariate analysis are shown in Supplementary Fig. 2a–d.

Figure 1c, d shows the disease-free survival (DFS) plots for both SNPs rs12268840 and rs9972882. Disease-free survival for SNP rs9972882 was worst in group AA (*n* = 14, median DFS = 13.5 months) compared with groups AC (*n* = 65, median DFS = 106.1 months) and CC (*n* = 111, median DFS = 90.1 months), though not significant (*p* = 0.0675). Likewise, there were no significant differences in disease-free survival curves of groups TT (*n* = 23, median DFS = 47.1 months), TC (*n* = 65, median DFS = 121.4 months), and CC (*n* = 101, median DFS = 90.1 months) of SNP rs12268840, with none of the groups showing any outstanding tendency according to the logrank test (*p* = 0.5625).

### Multivariate Regression

To further evaluate the significance of the identified SNPs, a multivariate regression analysis was performed including the clinical prognostic factors (Table 3). The histological parameters pT (*p* = 0.020) and pN category (*p* = 0.001) as well as R status (*p* = 0.006) were statistically significant as well as the SNPs rs12268840 (*p* = 0.033) and rs9972882 (*p* = 0.041). In a confirmative Cox regression model, the parameters pT category (*p* = 0.009), pN category (*p* < 0.001), R status (*p* < 0.001), rs12268840 (*p* = 0.045), and rs9972882 (*p* = 0.030) were significant survival predictors.

**Table 3:** Multivariate overall survival analysis, Cox regression, and successive confirmation

Predictor variable	Hazard ratio	SE	Lower 95% limit	Upper 95% limit	*p*-value
ASA score	1.36	0.29	0.90	2.06	0.147
Localization	1.07	0.14	0.83	1.39	0.583
Neoadjuvant therapy	1.42	0.44	0.77	2.62	0.259
pT category*	1.45	0.23	1.06	1.99	0.020
pN category**	1.48	0.17	1.18	1.86	0.001
pM category	1.46	0.53	0.72	2.98	0.293
R status**	2.51	0.84	1.31	4.84	0.006
Laurén type	1.09	0.18	0.79	1.52	0.595
rs12268840 (*MGMT*)*	1.47	0.26	1.03	2.08	0.033
rs9972882 (*STARD3*)*	0.66	0.13	0.44	0.98	0.041
rs12724079 *(ASH1L)*	1.10	0.20	0.76	1.58	0.616
rs10423674 *(CRTC1)*	1.30	0.34	0.78	2.17	0.306
rs1870377 *(VEGFR2)*	1.07	0.25	0.68	1.68	0.785
rs2898290 (*LINC00208*)	0.95	0.19	0.64	1.41	0.804
rs2976392 *(PSCA)*	1.09	0.21	0.75	1.58	0.649
rs2296616 *(miR-107)*	1.09	0.20	0.76	1.55	0.647
rs10419226 *(CRTC1)*	0.79	0.18	0.50	1.23	0.294
rs2701108 *(TBX5)*	0.80	0.17	0.52	1.22	0.306
rs3072 *(GDF7)*	1.42	0.33	0.90	2.23	0.134
rs4648068 *(NFKB1)*	1.33	0.28	0.88	2.01	0.173
rs7626449 *(DHSs1)*	0.98	0.20	0.66	1.45	0.915

pT category**	1.34	0.112	1.08	1.67	0.009
pN category***	1.45	0.089	1.22	1.73	< 0.001
R status***	2.69	0.264	1.61	4.51	< 0.001
rs12268840 (*MGMT*)*	1.33	0.142	1.01	1.75	0.045
rs9972882 (*STARD3*)*	0.71	0.157	0.52	0.97	0.030

^*^p < 0.05

^**^p < 0.01

^***^p < 0.001

### Characterization and Clinical Differences for rs12268840 (MGMT) and rs9972882 (STARD3)

Prognostic parameters such as the pT (*p* = 0.4966), pN (*p* = 0.1675), and pM category (*p* = 0.5984) or the R status (*p* = 0.6419) were not different between the three groups CC, TC, and TT for SNP rs12268840. However, we found that rates of second primary carcinomas (SPC) were significantly higher in the TT group (average rate 30.4%) as compared with 9.2% in the TC group and 4.0% in the CC group (*p* = 0.0003). Furthermore, the three groups differed significantly in preoperatively detected Laurén type (*p* = 0.0243).

In total, 42.9% of patients in group AA of SNP rs9972882 had distant metastases (pM1) on final histopathological workup as opposed to 12.3% in group AC and 13.5% in group CC (*p* = 0.0117). The remaining histological parameters pT (*p* = 0.1029) and pN category (*p* = 0.1226) as well as the R status (*p* = 0.6976) were not significantly different between the groups, either. All results for SNPs rs12268840 and rs9972882 are summarized in Supplementary Table 3A,B.

### Cis Expression Quantitative Trait Loci (cis-eQTL) in Normal Upper Gastrointestinal Tissue

To assess if the above-mentioned two SNPs were also able to change the level of gene expression, we further performed cis expression quantitative trait loci (cis-eQTL) analysis for all local genes in normal gastric and esophageal tissue (mucosa, muscularis, and gastroesophageal junction). The results are presented in Supplementary Table 4A,B.

**Fig. 2:**
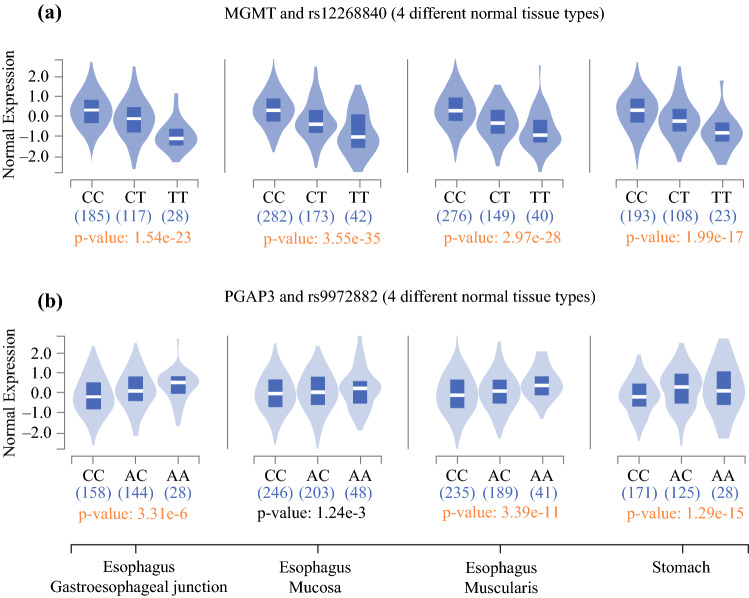
**a** Violin plots for *MGMT* expression and groups CC/CT/TT in rs12268840 in four different normal tissue types (gastroesophageal junction, esophageal mucosa, esophageal muscularis, and stomach). **b** Violin plots for *PGAP3* expression and groups CC/AC/AA in rs9972882 in the same four upper gastrointestinal tissues

We found that rs12268840 was a highly significant cis-eQTL for *MGMT* in all upper gastrointestinal tissue types. This is supported by the normalized effect size (NES) of −0.60 in gastroesophageal junction tissue (*p* = 1.5 × 10^−23^), meaning that the alternative allele (in this case, T) leads to a significantly lower expression of *MGMT* as compared with the reference allele C. Likewise, in esophageal mucosa NES was −0.64 (p = 3.5 × 10^−35^), in esophageal muscularis −0.52 (*p* = 3.0 × 10^−28^), and in gastric tissue −0.56 (*p* = 2.0 × 10^−17^) (Fig. 2a).

rs9972882 was a significant cis-eQTL for PGAP3 in normal gastric, gastroesophageal, and esophageal muscularis tissue (but not in esophageal mucosa). NES in gastroesophageal junction tissue was −0.15 (*p* = 3.3 × 10^−6^), in esophageal muscularis −0.17 (*p* = 3.4 × 10^−11^), and in gastric tissue −0.23 (*p* = 1.3 × 10^−15^). In all normal upper gastroesophageal tissue types, rs9972882 did not prove to be a significant eQTL for *STARD3* and *ERBB2* (*Her2/neu*) (Fig. 2b).

## Discussion

In this study we retrieved SNPs from the current literature that were reported to be associated with either BE, GERD, GEJ, or GC. We then analyzed theses SNPs via genotyped and imputed data of curatively resected patients at our medical center. We were able to show that two intron variant SNPs of *MGMT* and *STARD3* were significant predictors for overall survival but not for disease-free survival in gastroesophageal adenocarcinoma. Group TT of rs12268840 showed the highest rate of second primary carcinomas, lowest mRNA expression of *MGMT* in normal tissue, and worst oncologic outcome, implicating that rs12268840 could be involved in tumor progression. Group AA of rs9972882 had the highest rate of distant metastases (pM1), highest mRNA expression of *PGAP3*, and also worst outcome. The results are consistent with previous works and open a new perspective on clinically relevant germline SNPs that could directly affect carcinogenesis and tumor relapse in upper gastrointestinal cancer.

The SNPs that were selected by this literature-based approach have been identified as relevant for esophageal and gastric adenocarcinoma according to previous genome-wide association studies (GWAS) on mostly large collectives. Similar attempts of correlating SNPs with clinical outcome parameters were made by Sung et al. in 2017.^8^ The authors analyzed 11 SNPs that were previously identified as high-evidence genetic susceptibility markers for GC in a former meta-analysis.^9^ Sung et al. were not able to find significant associations of these 11 SNPs with overall survival. However, the authors found subtype-specific associations for GC of the cardia region and diffuse-type GC.

In our study, we confirmed usual prognostic factors such as pTNM category and R status on univariate survival analysis and all variables (except pM category) on multivariate analysis. Based on these results, we assume that this study’s validity is ensured concerning the identification of further survival predictors. Only two SNPs, namely rs12268840 (intron variant of *MGMT*) and rs9972882 (intron variant of *STARD3*), were significant on multivariate analysis and will therefore be further discussed. According to the Cox regression model, both SNPs were independent survival predictors next to pT/pN categories and R status.

SNP rs12268840 is located on chromosome 10 and is known as an intron variant of *MGMT* that encodes for the *O*-6-methylguanine-DNA methyltransferase (abbreviated as MGMT). DNA methylation is one of the most important types of epigenetic modification and plays a crucial role in carcinogenesis.^10^ MGMT is a DNA-repair enzyme that protects cells from carcinogenic effects by removing DNA adducts of alkylating agents from the *O*-6 position of guanine.^11^ Thus, studies have shown that loss of MGMT leads to an increased carcinogenic risk in mice.^12^ For gastric cancer, Zhang et al. were able to demonstrate that *MGMT* silencing via promoter hypermethylation was significantly associated with an increased risk of GC.^13^ Other works have shown that reduced expression of *MGMT* in esophageal adenocarcinoma organoids led to sensitivity towards temozolomide and taxane agents.^14^ The study by Doecke et al. that initiated the inclusion of rs12268840 to this literature-based approach found that homozygous carriers of rs12268840 (i.e., group TT) with frequent acid reflux had a significantly higher risk of GEJ.^15^

Our cis-eQTL analysis has shown that, in upper gastrointestinal tissues of healthy probands, group TT expressed the least MGMT mRNA in comparison with groups CC and CT (Fig. 2a). Furthermore, within the groups of rs12268840, there were significant different rates of second primary carcinomas (SPC). SPCs are defined as any type of malignant disease that was reported in the patient’s past medical history and was treated beforehand.^16^ Patients in group TT had the highest rate of SPC, while patients in group TC and CC had lower rates of SPC and significantly higher median OS. We interpret these results as group TT possibly having had a lack of MGMT to fight malignant diseases in the past medical history as well as in the postoperative follow-up. This may have led to a worse oncologic outcome in general.

Although SNP rs9972882 most likely qualifies as an intron variant of *STARD3*, it proved to be a significant cis-eQTL for *PGAP3* according to mRNA expression in normal upper gastrointestinal tissue. In contrast, rs9972882 was not a significant eQTL for neighboring genes *STARD3* or *ERBB2* (*Her2/neu*).

*PGAP3* transcribes the post-GPI attachment to proteins phospholipase 3, which is an enzyme for fatty acid remodeling of glycosylphosphatidylinositol (GPI)-anchored proteins (abbreviated as GPI-APs). GPI-APs are glycoproteins that are anchored to the outer layer of the plasma membrane and therefore exposed on the cell surface. Precisely, PGAP3 is responsible for the removal of unsaturated fatty acids at the sn-2 position of GPI-APs. Germline mutations of *PGAP3* lead to a subtype of hyperphosphatemia and intellectual disabilities, also known as Mabry syndrome type 4.^17^ Kwon et al.^18^ have found that *PGAP3* is co-amplified (together with *STARD3*, *GRB7*, and *MIEN1*) with *ERBB2(Her2/neu)* in esophageal and gastric cancer. Murakami et al.^19^ were able to show that *PGAP3* knockout mice had enhanced responses to alloreactive and antigen-specific stimuli. It is clear that GPI-APs play important roles in many biological processes such as signal transduction and cell–cell interaction. Our data could be interpreted that overexpression of *PGAP3* within group AA of rs9972882 represents a malfunctioning of GPI anchoring.

The classification of gastric cancer by The Cancer Genome Atlas (TCGA) Research Network into Epstein–Barr virus (EBV)-positive, microsatellite instability (MSI)-high, genomically stable (GS), and chromosomally instable (CIN) subtypes has drawn attention to a general molecular differentiation of gastric cancer.^20^ The differentiation of molecular subtypes could be especially relevant for the prediction of potential susceptibility for (neo-)adjuvant treatment.^21^ So far, the CIN subtype was encountered more frequently in the gastroesophageal junction and cardia,^22^ which was also the most frequent anatomic location of gastric adenocarcinoma in this study group. Due to its role as a DNA-repair enzyme, MGMT and its dysfunction may represent another aspect of the CIN subtype demonstrated by TCGA. However, tumor tissue and sequencing patterns would be necessary to properly investigate the effect of MGMT expression (and PGAP3, respectively) on actual prognosis for the separate TCGA subtypes. For this reason, genomic and tumor-specific data analysis will be necessary to infer that these results are generally applicable to all gastroesophageal cancer subtypes.

This study is based on a data collection of numerous clinical variables to enable a profound analysis of essential prognostic factors accompanied by reliable survival data from a systematic follow-up. Moreover, our analyses allow a linkage and translational approach to investigate histopathological, oncological, and genotyped features of upper gastrointestinal tumors. The ultimate goal of this study was to identify those genetic alterations that actually have a clinical implication in terms of a significantly different prognosis. However, it is necessary to remark that there may be a potential selection bias concerning those SNPs that could be of interest but were not investigated due to missing reports in the literature. Another limitation of this study is that both GEJ and GC were analyzed in the same collective in terms of testing for specific SNP markers. However, we were able to prove beforehand that both groups did not differ in decisive outcome parameters. Last but not least, the separate analysis of both groups GEJ and GC did not show any significant differences, neither for overall nor for disease-free survival. Previous works by our own group have demonstrated the comparability of both cancer entities regarding long-term oncological outcome.^23^ This circumstance may also be in accordance with the insights by TCGA that distant esophageal adenocarcinoma and chromosomally unstable gastric adenocarcinoma (the most frequent TCGA subtype of gastric cancer) share many genomic amplifications and could possibly be considered a single disease entity.^24^

By correlating the above-mentioned SNPs with our clinical data, we identified two clinically relevant SNPs. Our data suggest that the DNA-repair enzyme MGMT is relevant in gastroesophageal cancer and that PGAP3 is a potentially novel agent in carcinogenesis. In the future, it will be necessary to reproduce these results for larger and more homogeneous samples. Eventually, in vitro studies are required to comprehend the molecular mechanisms of *MGMT* and *PGAP3*.

## Supplementary Information

Below is the link to the electronic supplementary material.

Supplementary file1 (JPG 211 kb)

Supplementary file2 (JPG 375 kb)

Supplementary file3 (XLSX 63 kb)
