# Supplementary figures and images for: Clinical Relevance of Gastroesophageal Cancer Associated SNPs for Oncologic Outcome After Curative Surgery
Source: Ann Surg Oncol. 2021 Sep 16;29(2):1453–62. doi: 10.1245/s10434-021-10771-y (PMC8724221; doi:10.1245/s10434-021-10771-y)

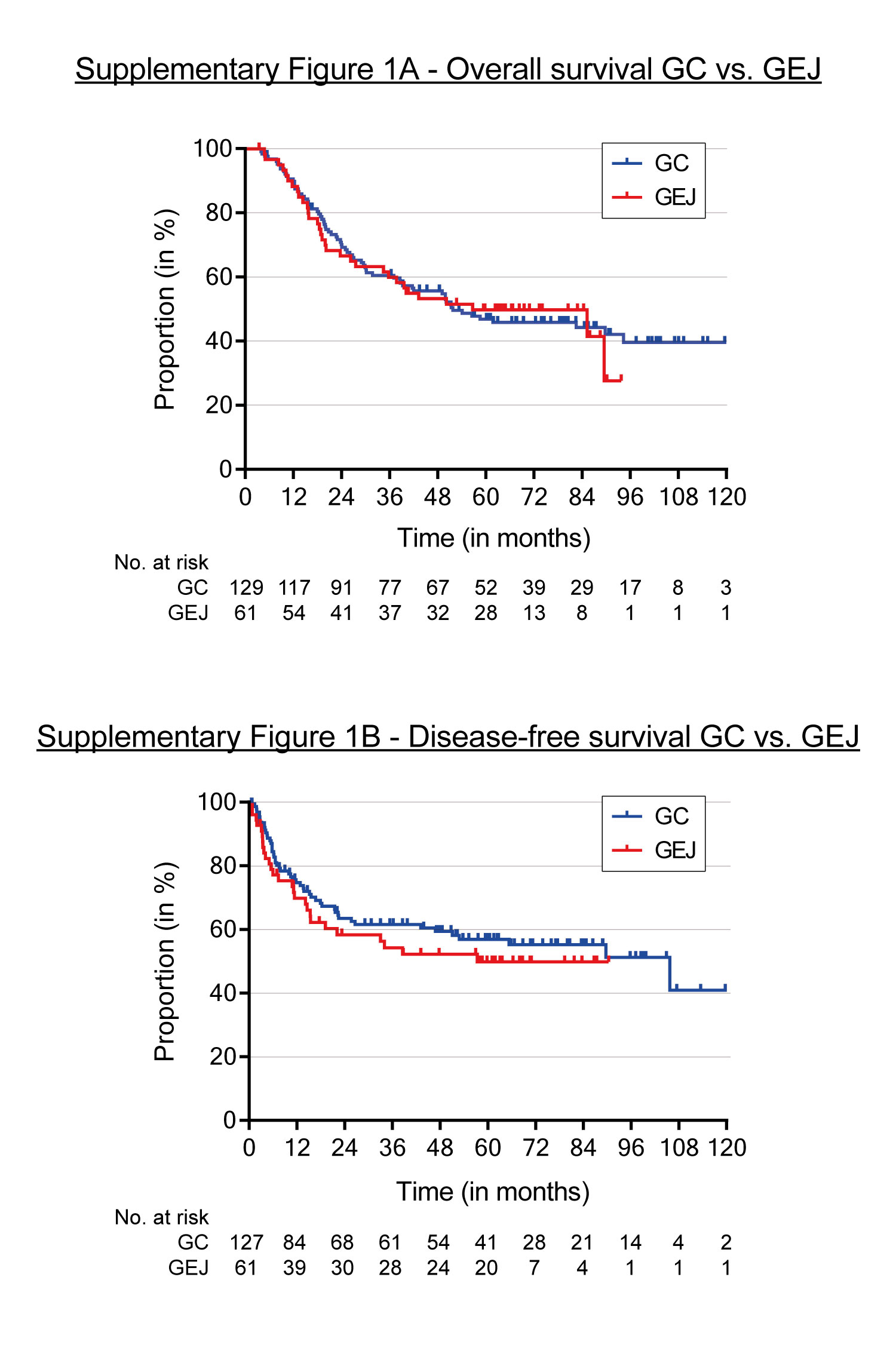

Supplement: Supplementary file 1 — Supplementary file1 (JPG 211 kb) [file 10434_2021_10771_MOESM1_ESM.jpg]

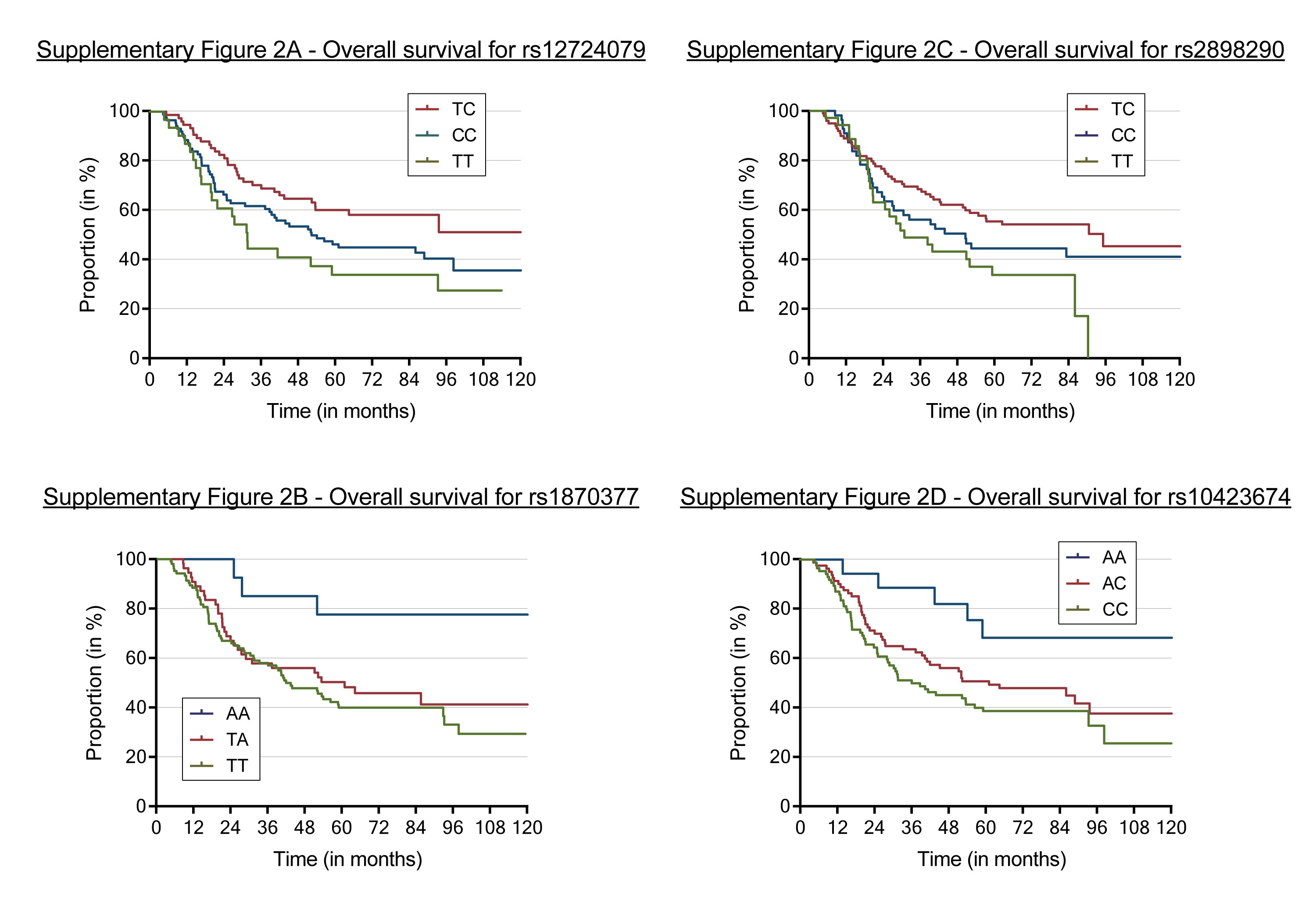

Supplement: Supplementary file 2 — Supplementary file2 (JPG 375 kb) [file 10434_2021_10771_MOESM2_ESM.jpg]
